# Effect of Dance Intervention on Cognitive Function and Related Cerebellar Dynamic Functional Connectivity in Patients With Schizophrenia

**DOI:** 10.31083/AP45310

**Published:** 2026-04-20

**Authors:** Hui He, Gujing Li, Yuanyuan Yu, Yayun Liu, Tianhuan Li, Kexin Gao, Ping Xi, Frank Pollick, Jing Lu, Lixue Yin, Dezhong Yao, Li Mi, Cheng Luo

**Affiliations:** ^1^The Clinical Hospital of Chengdu Brain Science Institute, School of Life Science and Technology, University of Electronic Science and Technology of China, 611731 Chengdu, Sichuan, China; ^2^The Clinical Hospital of Chengdu Brain Science Institute, Chengdu Mental Health Centre, The Fourth People’s Hospital of Chengdu, 610036 Chengdu, Sichuan, China; ^3^Cardiovascular Ultrasound and Non-invasive Cardiology Department, Sichuan Provincial People’s Hospital, 610072 Chengdu, Sichuan, China; ^4^School of Psychology and Neuroscience, University of Glasgow, G2 1AL Glasgow, UK; ^5^Cuban Neuroscience Center, 10000 La Habana, Cuba; ^6^China-Cuba Belt and Road Joint Laboratory on Neurotechnology and Brain-Apparatus Communication, University of Electronic Science and Technology of China, 611731 Chengdu, Sichuan, China

**Keywords:** dance intervention, cerebellum, schizophrenia, functional magnetic resonance imaging, cognition

## Abstract

**Background:**

The cerebellum is a critical node implicated in the pathology of schizophrenia. Previous studies have demonstrated that dance interventions can enhance cerebellar functional connectivity in healthy individuals. These findings suggest that dance intervention might represent a promising treatment for patients with schizophrenia.

**Methods:**

A total of 32 patients with schizophrenia were randomly assigned to two groups: a dance group (n = 18) and an aerobic exercise group (n = 14). Clinical symptoms and cognitive function, along with resting-state functional magnetic resonance imaging (fMRI) data, were collected from all participants at baseline and post-intervention. The cerebellar motor (CBCm) and cerebellar cognitive (CBCc) regions were defined for the calculation of dynamic functional connectivity (dFC). Repeated-measures analyses of covariance (ANCOVAs) were conducted for statistical analysis.

**Result:**

Significant interaction effects were observed for cognitive function and dFC. Dance intervention specifically enhanced cognitive function, as assessed by the Continuous Performance Test-Identical Pairs (CPT-IP) and the Hopkins Verbal Learning Test-Revised (HVLT-R). Moreover, increased dFC was observed between the CBCm and the left medial superior frontal gyrus, left superior occipital gyrus, and right cuneus, as well as between the CBCc and the right median cingulate cortex and the left inferior temporal gyrus in the dance intervention group. Additionally, dFC between the CBCc and the median cingulate gyrus showed a positive correlation with CPT-IP (r = 0.412, *p* = 0.026) and HVLT-R (r = 0.414, *p* = 0.021) scores.

**Conclusions:**

These findings suggest that dance intervention specifically enhances cerebellar connectivity patterns, potentially improving attention and verbal memory in patients with schizophrenia.

**Clinical Trial Registration:**

No: ChiCTR2100049273, https://www.chictr.org.cn/showproj.html?proj=65597.

## Main Points

1. Dance training improves attention and verbal memory in schizophrenia.

2. Dance training promotes cerebellar plasticity in schizophrenia.

3. Cognitive gains are associated with dynamic cerebellar cognitive (CBCc)–cingulate connectivity.

## 1. Introduction

Schizophrenia is a serious psychiatric disorder, affecting about 0.4% of the population, with a lifetime risk estimated at 7.2% [1], placing a significant burden on both society and individuals [2, 3]. Prolonged treatment with antipsychotic drugs is a common choice for the remission stage of schizophrenia. Complementary therapies, such as cognitive-behavioral intervention, are also used for such patients. As an adjunctive treatment for schizophrenia, dance intervention has been shown to effectively improve both clinical symptoms and cognitive functioning [4, 5]. The specific mechanisms behind this phenomenon, however, are still poorly understood.

Dance intervention comprises a series of elaborate technical practices that facilitate the systematic processing and integration of diverse internal and external sensorimotor information. Specifically, dance integrates complex motor sequences, rhythm perception, balance, and visuospatial coordination, all of which require sustained attention, working memory, and executive control [6]. Moreover, dance has also been regarded as an effective non-pharmacological complementary treatment for older adults [7], people with mild cognitive impairment [8, 9], and schizophrenic subjects [10]. Aerobic exercise shares several similarities with dance, such as coordinated full-body movement and comparable physical activity. Many studies have shown that physical exercise has positive effects on both healthy individuals and schizophrenic subjects [11, 12, 13]. However, other research has shown that dance intervention exerts a more pronounced effect on brain plasticity in older adults compared to repetitive physical exercise [14], suggesting that dance may represent a particularly promising approach in improving cognitive deficits of schizophrenia.

It is proposed that the cerebellum plays a key role in mediating the effects of dance intervention on brain plasticity. A previous study has demonstrated that prolonged dance intervention enhances cortico-basal ganglia loops in professional dancers [15]. The cerebellum is elaborately connected with the basal ganglia and cerebral cortex, forming a cohesive and integrated neural network [16]. Additional study has revealed that a 6-month dance intervention enhanced functional connectivity (FC) between cerebellar and visual networks in older adults [17]. Importantly, the cerebellum is closely linked to motor coordination, perceptual processing, and higher-order cognitive functions [18, 19], has been identified as a central pathological brain region in schizophrenia. Evidence from anatomical [20], functional neuroimaging [21], and molecular [22] studies highlights the presence of cerebellar dysfunctions in sensorimotor and cognitive processing in schizophrenia [21]. Dysfunctions have been identified in the prefrontal-thalamic-cerebellar circuitry in schizophrenia, where the cerebellum coordinates both motor and cognitive performance and interacts dynamically with the prefrontal cortex [23]. Reduced FC has been reported between the cerebellum and language-related regions [24]. In a recent investigation of the principal gradient of cerebellar FC in schizophrenia, it was concluded that disruptions in low-level sensorimotor systems may partially account for high-level cerebellar dysfunction [25]. Overall, these results indicate that the cerebellum may provide a key neural basis subserving the beneficial effects of dance intervention in individuals with schizophrenia.

Based on previous studies, the cerebellum is hypothesized to provide a critical node in facilitating improvements in schizophrenia through dance intervention. Compared to resting-state FC, dynamic functional connectivity (dFC) analysis offers a more comprehensive understanding of FC alterations in schizophrenia [26]. In a previous study, the current authors identified the value of dance intervention in improving negative symptoms and cognitive deficits, including sustained attention and verbal memory in patients diagnosed with schizophrenia [27]. This study aimed to further examine neural mechanisms underlying the effects of dance intervention in patients with schizophrenia. Two cerebellar subregions were defined as regions of interest (ROI), for examination of alterations in cerebellar dFC following a three-month dance intervention. An aerobic exercise group with matched intensity and duration was included to control for the general effects of physical activity. It was hypothesized that the dance intervention would increase cerebellar dFC and that such neural changes would relate to enhanced cognitive performance.

## 2. Methods and Materials

### 2.1 Subject Information

The study initially recruited 60 patients with schizophrenia from the Fourth People’s Hospital of Chengdu. All individuals were required to have been on a stable antipsychotic regimen for at least eight weeks prior to and throughout the intervention period. Inclusion criteria included: (1) a confirmed diagnosis of schizophrenia according to the Diagnostic and Statistical Manual of Mental Disorders, Fourth Edition (DSM-IV) standards; (2) age between 30 and 60 years; and (3) absence of any suicide attempts within the preceding six months [28]. Exclusion criteria included: (1) presentation of acute-phase schizophrenia or symptoms indicative of an onset episode; (2) presence of organic brain pathology; (3) severe or decompensated chronic medical conditions; (4) body weight falling outside the normal range (overweight or underweight); (5) notable cardiovascular, neuromuscular, endocrine, or other systemic disorders; (6) marked claustrophobic tendencies; and (7) insufficient cognitive ability to understand or follow the study procedures.

### 2.2 Physical Activity Intervention Procedures

**Fig. 1.**
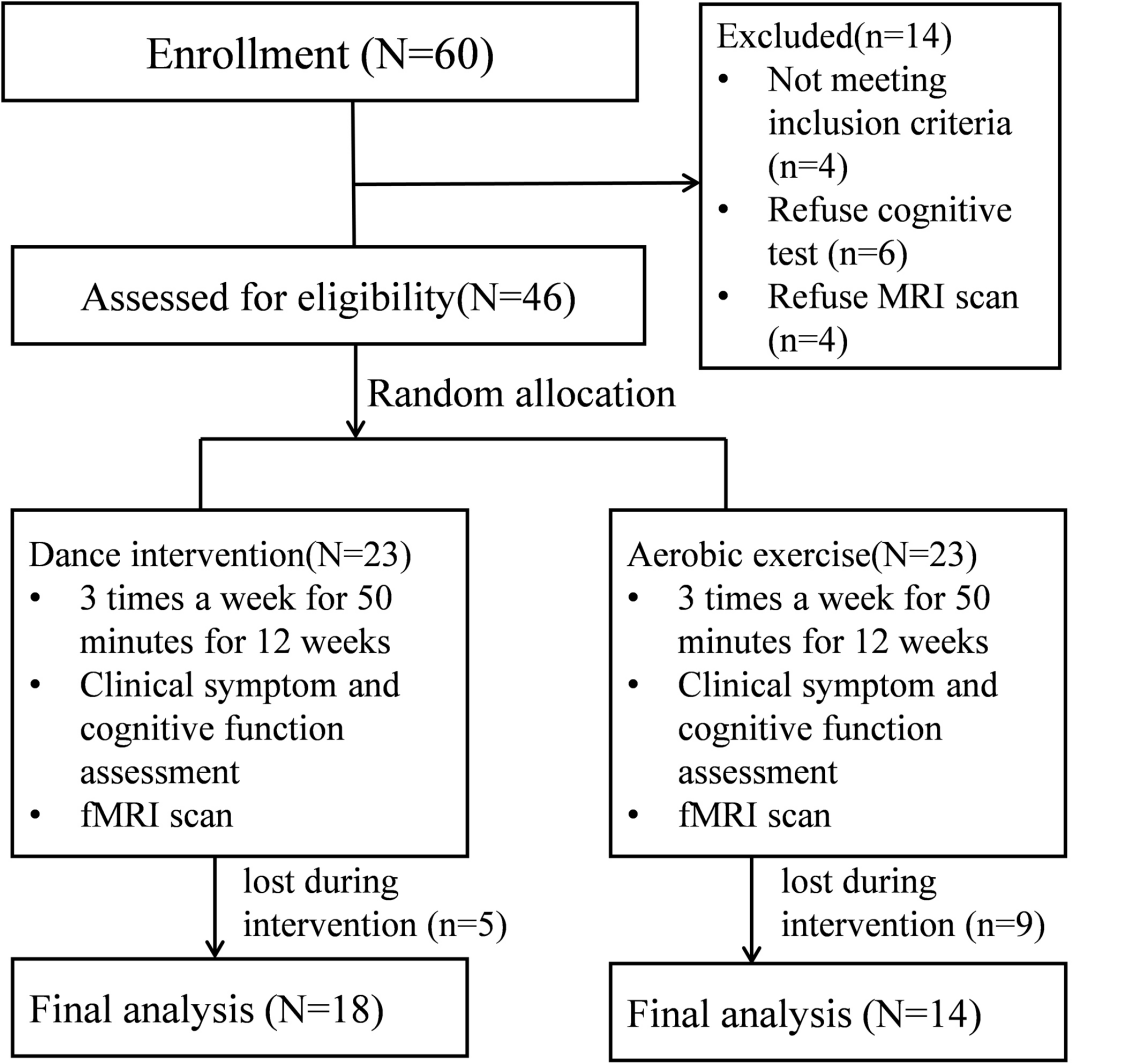
**Patient flow in the dance intervention group and aerobic exercise group**. fMRI, functional magnetic resonance imaging.

The study was a clinical trial in which subjects were screened and then randomly assigned to either a dance intervention group or an aerobic exercise control group. To minimize potential confounding factors, all subjects continued receiving only standard medication throughout the study. The specific patient flows were shown in Fig. 1. The detailed experimental design and behavioral results have been reported in a previous publication [27]. The specific information can be found in **Supplementary Material A,B**. The current study focused on the alterations in dFC following the intervention. The intervention procedures are now briefly summarized:

(1) Recruitment and Screening of Subjects: sixty patients were recruited and screened based on predefined inclusion and exclusion criteria.

(2) Baseline Data Collection: before the experiment, subjects completed a series of baseline assessments. Clinical symptoms were evaluated using the Positive and Negative Symptom Scale (PANSS) [29]. Cognitive performance was measured with the MATRICS Consensus Cognitive Battery (MCCB) [30]. Additionally, all subjects underwent functional magnetic resonance imaging (fMRI) scans. The study implemented a double-blind design, ensuring that both assessors and subjects were blinded to group assignments until the experiment commenced. The baseline information of the scale can be found in Table 1 and **Supplementary Material C**.

**Table 1.** **Comparison of baseline differences in Demographic variables**.

	Dance intervention group	Aerobic exercise group	*p*
Gender (Female/Male)	10/8	5/9	0.49^a^
Age (years)	50.00 (6.95)	51.00 (8.47)	0.90^b^
Education level (years)	11.11 (3.25)	10.36 (2.62)	0.18^b^
Weight (kg)	63.17 (9.09)	64.93 (11.93)	0.73^b^
Height (cm)	162.61 (6.62)	164.14 (6.26)	0.79^b^
BMI (kg/m^2^)	23.79 (2.50)	24.21 (4.90)	0.67^b^
Duration of illness (years)	23.44 (9.17)	25.00 (9.91)	0.65^b^
Onset age	27.06 (7.68)	26.00 (7.58)	0.70^b^
Medication dosage in CPZ equivalents (mg)	316.9 (142.83)	423.1 (181.37)	0.09^b^
Number of Intervention sessions	38.00 (7.25)	37.36 (7.96)	0.81^b^

Indicated values are shown as mean (standard deviation). ^a^*p* values for the comparisons (chi-square test) between the dance group and the aerobic exercise group; ^b^*p* values for the comparisons (independent-*t* test) between the dance group and the aerobic exercise group. BMI, body mass index; CPZ, chlorpromazine.

(3) Randomized Group Assignment: following randomization principles, subjects were allocated to either the dance or aerobic exercise intervention group. No significant differences were found between the groups with regard to demographic variables, clinical symptoms, or cognitive performance.

(4) Implementation of the Intervention: subjects in the dance intervention group joined a structured dance training program. Those in the aerobic exercise group took part in standardized physical exercise sessions. Both interventions were conducted over a 12-week period, with three 50-minute sessions per week. (a) Dance intervention group: the program emphasized observation and imitation of dance movements, as well as learning, memorizing, and synchronizing actions with musical rhythms. The training aimed to enhance body awareness and control. The dance sequences were quantitatively structured along the dimensions of time, space, and intensity, with each movement assigned a specific meaning to facilitate subject comprehension. (b) Aerobic exercise program: the program was simplified to include only basic physical movements, thereby minimizing the cognitive demands on the subject. The intensity and volume of exercise were assessed via wrist-worn devices, and no significant group differences were detected.

(5) Post-intervention Data Collection: when the interventions were finished, all subjects underwent follow-up magnetic resonance imaging (MRI) scans and were reassessed for clinical and cognitive outcomes. The same personnel conducted assessments using consistent methods as those employed before the intervention. For further details on the experimental design, please refer to our previous research [27].

### 2.3 fMRI Data Collection and Preprocessing

Resting-state fMRI images were collected on a 3.0 T scanner (Siemens, Erlangen, Germany). During scanning, foam padding and earplugs were used to minimize head motion and reduce scanner noise. Functional images were obtained using a gradient-echo echo-planar imaging (EPI) sequence (repetition time: 2 s, echo time: 30 ms, flip angle: 90°, matrix size: 64 × 64, field of view: 240 × 240 mm^2^, slice thickness/gap: 4/0.4 mm) with an eight-channel phased-array head coil. Each patient completed a 510-second resting-state scan, yielding a total of 255 volumes.

Functional image preprocessing was performed in DPABI (V8.2-240510, DPARSF 5.4, http://rfmri.org/dpabi) [31] following standard procedures. The initial five volumes were discarded. Preprocessing included slice-timing correction, realignment, normalization to Montreal Neurological Institute (MNI) space, and smoothing with a 6 mm full width at half maximum (FWHM) Gaussian kernel. Nuisance regression incorporated Friston-24 motion parameters, white matter and cerebrospinal fluid signals, and linear trends. Volumes exhibiting framewise displacement >0.2 mm (Jenkinson method) were treated as outliers and included as separate regressors. Global signal regression was not performed. The fMRI data were filtered within a frequency range of 0.01–0.08 Hz.

### 2.4 ROI Based Dynamic Functional Connectivity

Two clusters within the cerebellum were defined as our ROIs using the human cerebellum atlas [18]. DFC was estimated using a sliding-window method implemented in DynamicBC v2.2 (University of Electronic Science and Technology of China, Chengdu, Sichuan, China) [32]. Hamming windows (width: 50 repetition times [TRs]; step size: 1 TR) were applied to the blood oxygen level dependent (BOLD) time series, sliding sequentially across the data [33]. In total, 250 time points were divided into 201 time windows. Pearson correlation was used to compute the relationship between the time series of the two ROIs and all brain voxels within each time window. Fisher Z-transformation was subsequently performed and the standard deviation of the Z-scores across all windows was calculated to obtain the dFC.

### 2.5 Statistical Analyses

Differences in dFC were examined using repeated-measures analyses of covariance (ANCOVAs) in DPABI, controlling for age, gender, education, height, and weight. The model included time (baseline vs. post-treatment) and group (dance vs. aerobic exercise) as factors, with dFC as the dependent variable. Multiple comparisons were corrected using Gaussian random field correction (*p* < 0.005 at the voxel level, *p* < 0.05 at the cluster level) [34]. For regions showing significant interactions, dFC values were extracted for post-hoc tests: paired-sample *t*-tests assessed within-group change and independent-sample *t*-tests compared groups at each time point.

A prior study reported that both dance intervention and aerobic exercise improved cognitive performance, with dance intervention yielding greater gains in sustained attention and verbal memory [27]. Based on these findings, the current analysis focused on FC and MCCB domains showing significant time × group interactions to explore underlying neural correlates. Spearman’s rank correlation was applied to assess relationships between changes in cognitive performance and alterations in cerebellar dFC (Δ = post – pre). All post-hoc and correlation analyses were conducted in MATLAB 2020b (MathWorks, Natick, MA, USA).

## 3. Results

### 3.1 Patients Demographics

32 patients remained for the final analysis, including 18 in the dance intervention group and 14 in the aerobic exercise group. There were no significant differences in the demographic variables (Table 1).

### 3.2 Effect of Dance Intervention on Cognitive Function

**Fig. 2.**
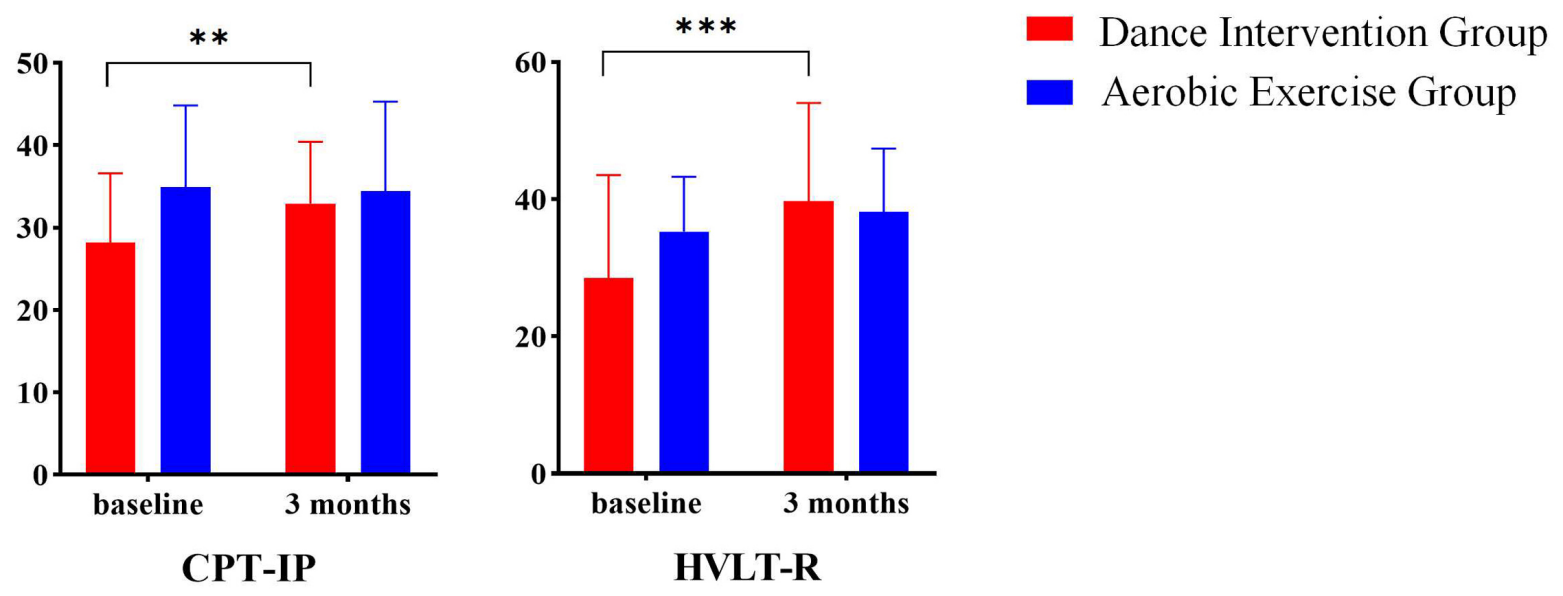
**Time × group interaction effect on cognitive function**. HVLT-R, Hopkins Verbal Learning Test-Revised; CPT-IP, Continuous Performance Test-Identical Pairs; **p < 0.005; ***p < 0.001.

A previous study has identified significant time × group interactions in the subscales of the MCCB, including the Continuous Performance Test-Identical Pairs (CPT-IP) and the Hopkins Verbal Learning Test-Revised (HVLT-R) scales (Fig. 2). The CPT-IP assesses sustained attention, while the HVLT-R evaluates verbal memory capacity. Other results related to MCCB are given in **Supplementary Material D**.

### 3.3 Effect of Dance Intervention on Dynamic Functional Connectivity

**Fig. 3.**
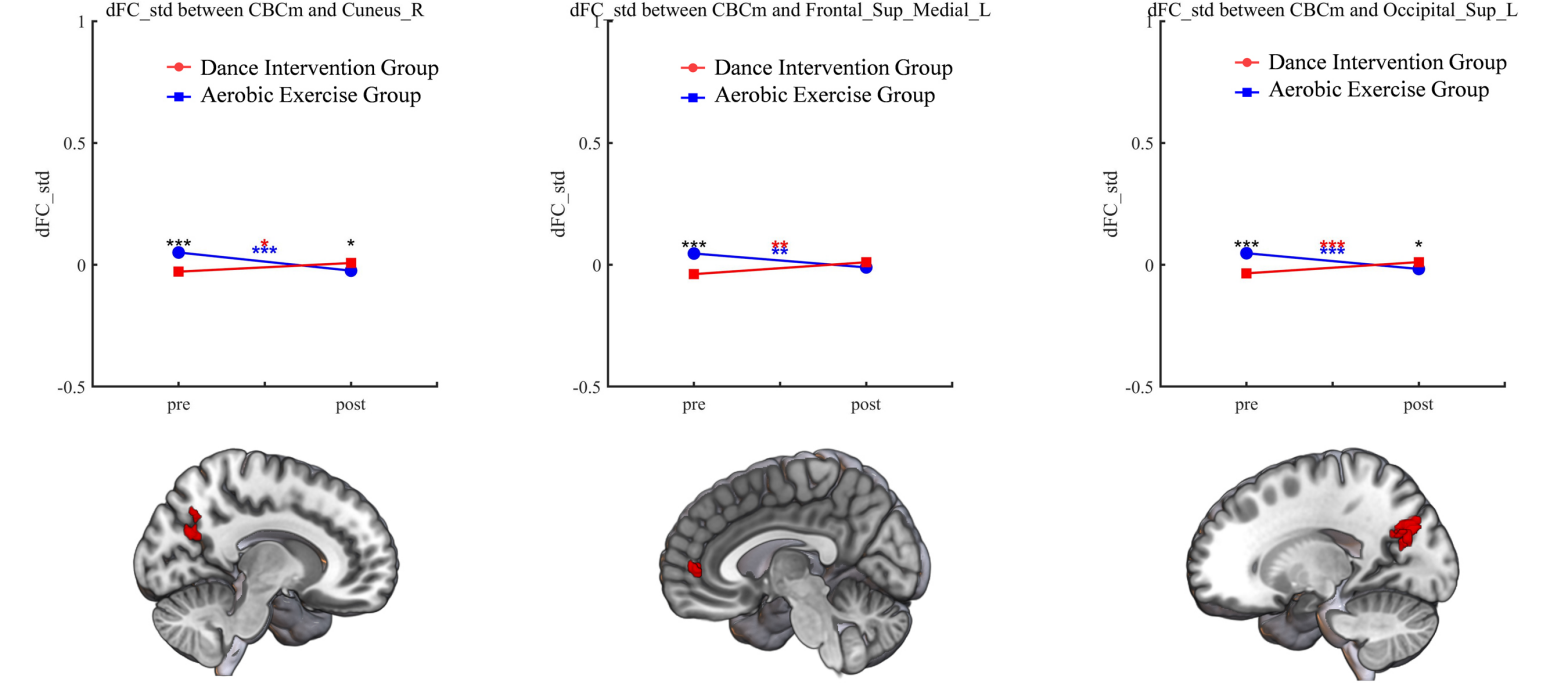
**Significant group × time interaction effect of CBCm**. *p < 0.05, **p < 0.01, ***p < 0.001. dFC, dynamic functional connectivity. Black asterisks (*) indicate significant within-group (pre–post) differences in both groups. Red and blue asterisks indicate significant pre–post differences in the dance intervention and aerobic exercise groups, respectively. Red-colored brain regions denote areas with significant differences.

**Fig. 4.**
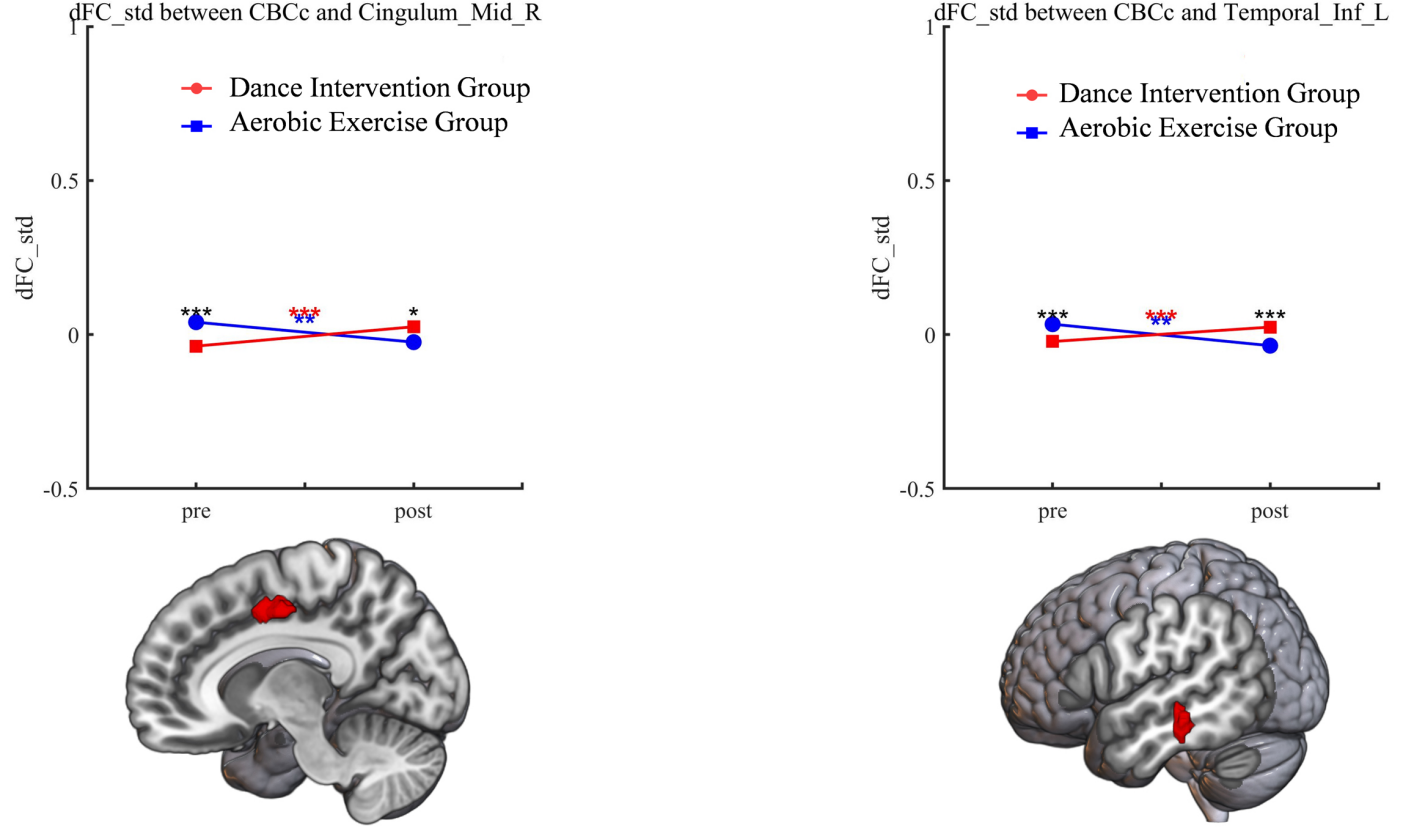
**Significant group × time interaction effect of CBCc**. *p < 0.05, **p < 0.01, ***p < 0.001. Black asterisks (*) indicate significant within-group (pre–post) differences in both groups. Red and blue asterisks indicate significant pre–post differences in the dance intervention and aerobic exercise groups, respectively. Red-colored brain regions denote areas with significant differences.

**Fig. 5.**
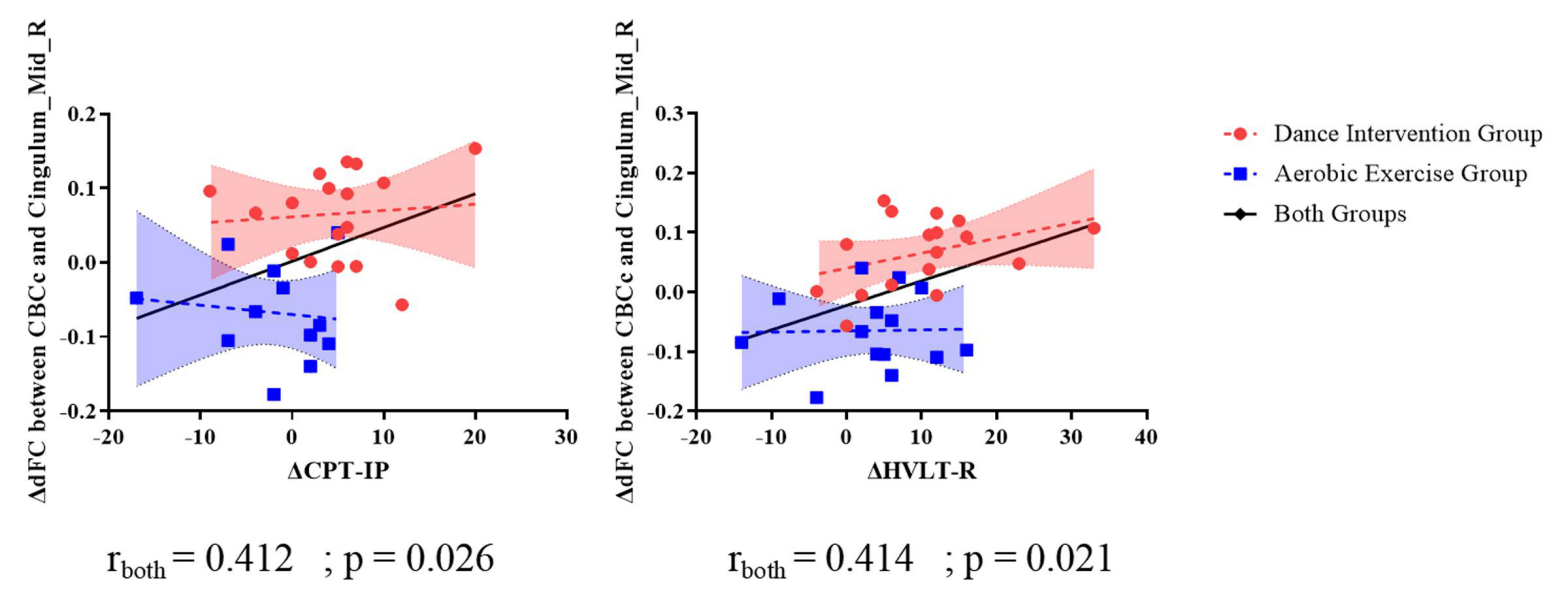
**Correlation between dFC and cognitive scale scores**.

Significant time × group interactions of dFC were observed between the cerebellar motor (CBCm) and left medial superior frontal gyrus (mSFG, Table 2, Fig. 3); and between the left superior occipital gyrus (SOG, Table 2, Fig. 3) and the right cuneus gyrus (CUN, Table 2, Fig. 3). The interaction effects of dFC were also found between cerebellar cognitive (CBCc) and the right median cingulum cortex (MCC, Table 2, Figs. 4,5) and left inferior temporal gyrus (ITG, Table 2, Fig. 4).

**Table 2.** **Results of Repeated-Measures ANCOVAs for Dynamic Functional Connectivity**.

ROI	Brain region	MNI coordination			Voxel size	F value
		x	y	z		
CBCm	Occipital_Sup_L	–21	–78	30	77	4.46
	Frontal_Sup_Medial_L	–6	54	6	28	3.37
	Cuneus_R	18	–63	36	27	3.70
CBCc	Cingulum_Mid_R	3	–9	45	74	4.31
	Temporal_Inf_L	–60	–30	–21	51	3.58

CBCm, cerebellar motor; Occipital_Sup_L (SOG), left superior occipital gyrus; MNI, Montreal Neurological Institute; ROI, regions of interest; Frontal_Sup_Medial_L (mSFG), left medial superior frontal gyrus; Cuneus_R (CUN), right cuneus; CBCc, cerebellar cognitive; Cingulum_Mid_R (MCC), right median cingulum cortex; Mid, median; Temporal_Inf_L (ITG), left temporal gyrus.

### 3.4 The Relationship Between dFC and Cognitive Function

Positive correlations were observed between the dFC (CBCc-medial cingulum gyrus) and the CPT-IP (r = 0.412, *p* = 0.026), as well as HVLT-R (r = 0.414, *p* = 0.021) in subjects across both groups. However, no such correlation was discovered in either the dance intervention group or the aerobic exercise group alone. For detailed results, refer to Fig. 5.

## 4. Discussion

This study explored how dance intervention affected the dFC of the cerebellar motor and cognitive modules. It also examined how these neural changes related to cognitive performance in schizophrenic subjects. Interaction effects were observed between the CBCm and the left mSFG, left SOG, and CUN, as well as between the CBCc and the right MCC and left ITG. A contrasting pattern emerged between the groups: dFC increased significantly in the dance intervention group but decreased markedly in the aerobic exercise group. Moreover, the dFC between the CBCc and right MCC was positively correlated with CPT-IP and HVLT-R scores, suggesting that stronger connectivity was associated with better cognitive performance. These findings indicate that dance intervention may promote specific cognitive improvements in individuals with schizophrenia.

### 4.1 Enhanced Visual Functional Connectivity Through Dance Intervention in Patients With Schizophrenia

Previous research indicated that patients with schizophrenia have an impaired visual system [35]. Reduced gray matter density in the occipito-temporo-frontal circuitry was associated with visual information processing in schizophrenia, encompassing the left SOG, left CUN, left SFG, and the left cerebellum [36]. Previous research has also reported reduced intrinsic neural timescales in the SOG region in schizophrenia, suggesting that sensory information in visual areas is retained for shorter durations compared to healthy controls [37].

Patients with schizophrenia also show widespread connectivity disturbances in the CUN, characterized by decreased dFC between the CUN and the middle temporal gyrus [38]. Another study found that FC in the left CUN in individuals with schizophrenia is negatively correlated with disease duration; that is, a longer disease duration is associated with reduced connectivity in the CUN lobe [39]. The observed dysfunction within the Visual Network (VN) may underlie the impaired visual perception seen in schizophrenia, which could further account for deficits in visual cognitive processes such as object recognition, face processing, and reading [40, 41]. In this study, schizophrenic subjects showed increased dFC between the motor cerebellum and the VN after three months of dance intervention. Dance is an activity that integrates visual, auditory, and kinesthetic information. Dance intervention accordingly strengthens visual pathways by requiring continuous monitoring of body position, spatial orientation, and external cues [42]. The observed increase in dFC could indicate that dance intervention facilitates greater functional coordination within the visual system.

### 4.2 Enhancement of Attention System in Patients With Schizophrenia Through Dance Intervention

In the dance intervention group, dFC between the CBCm and left mSFG showed a significant increase. A similar increase was also found between the CBCc and the right MCC and left ITG. All these regions are integral components of the attention system. The SFG has been implicated in goal-directed attentional processes and plays a pivotal role in spatial orientation processing [43, 44]. The anteromedial portion of the SFG contributes to cognitive control and is both anatomically and functionally linked to the MCC [45]. The posterior portion of the MCC is a confirmed component of the attention system [46] and plays a critical role in orienting vision, a function central to attentional control [47]. The ITG is a critical node in the attention network, facilitating object selection and engaging in object-based attentional processes [48].

Disruptions in the attention system have been well-documented in schizophrenia, particularly deficits in attentional control and integration [49, 50]. Dysfunction of the attention system in schizophrenia is further associated with impaired cognitive functions, including deficits in working memory, episodic memory, and sustained attention [51, 52]. In this study, increased dFC between these regions and the cerebellum was observed in the dance intervention group, which may be associated with the specific attentional demands inherent in dance practice. Engaging in dance requires sustained focus on complex movement sequences, synchronization with rhythm, and continuous monitoring of body position in space [53], potentially reflecting an enhancement in the attention system of dance intervention in patients with schizophrenia.

### 4.3 Enhanced dFC Correlates With Improved Cognition in Schizophrenia

The change in dFC between the CBCc and right MCC showed a positive correlation with CPT-IP and HVLT-R scores. Although no significant correlation was observed in either the dance intervention group or the aerobic exercise group, the trends in dFC between the two groups were contrasting, with increased dFC in the dance intervention group and decreased dFC in the aerobic exercise group.

Previous study has highlighted that attentional deficits are a core feature underlying perceptual abnormalities in schizophrenia [54]. Consistently, other studies have also reported impairments in verbal memory among individuals with schizophrenia [55, 56]. Attention is a fundamental cognitive process that plays a crucial role in higher-order cognitive functions, including verbal memory [57, 58]. This suggests that the increased dFC observed in the dance intervention group may reflect the capacity of dance to promote cerebellar-cortical plasticity and facilitate motor-cognitive integration. This interpretation aligns with previous evidence showing that dance training can induce structural and functional brain plasticity within cerebellar–cortical and sensorimotor networks [14, 17, 59, 60].

Additionally, the affective and embodied aspects of dance may contribute to language-related processing. The enjoyment of dance has been linked to improved language comprehension, as the understanding of dance movements can enhance the interpretation of verbal meanings [61]. During the process of dance learning, patients must execute complex movement sequences by accurately reproducing observed movements, a process that may involve converting both visual and verbal information into motor actions [62]. The MCC plays a key role in attention regulation, and the cerebellum contributes to language processing at both motor and cognitive levels [63, 64]. Thus, the increased dFC observed in the dance group may reflect improved attention and verbal function.

### 4.4 Limitations

This study is subject to several limitations: (1) The extended duration of the study, coupled with a high attrition rate, resulted in a relatively small sample size for analyses. Consequently, no significant association was observed between cerebellar dFC and clinical symptom improvement, possibly due to the small sample size. (2) Without a matched control group, it is hard to tell whether the changes resulted from the intervention or from the passage of time. (3) The intervention structures of the dance and aerobic exercise groups were not identical, which may have also influenced the outcomes. Additionally, the relatively small cluster sizes observed in the interaction effects, although surviving cluster-level correction, should be interpreted with caution. (4) The sample included only middle-aged patients, which limits the generalizability of our findings to younger populations. Future research should examine whether similar effects can be observed in younger patients with schizophrenia.

## 5. Conclusions

This study showed that a 3-month dance intervention specifically enhanced the dFC of the cerebellum in schizophrenic subjects. This suggests that dance intervention could enhance higher-order cognitive functions, including attention and verbal memory, by modulating the visual network and attention system in schizophrenic subjects.

## Availability of Data and Materials

The datasets used and analyzed during the current study are available from the corresponding authors on reasonable request.

## Supplementary Material

Supplementary material associated with this article can be found, in the online version, at https://doi.org/10.31083/AP45310.
