# Supplementary material for: Effect of Dance Intervention on Cognitive Function and Related Cerebellar Dynamic Functional Connectivity in Patients With Schizophrenia
Source: Alpha Psychiatry. 2026 Apr 20;27(2):45310. doi: 10.31083/AP45310 (PMC13156061; doi:10.31083/AP45310)
Supplement: Supplementary file 1 [file 2757-8038-27-2-45310-s1.zip › Supplementary Material.docx]

**Supplementary Material A**

To enhance patient motivation and minimize dropout rates, we introduced the two interventions in a supportive and engaging way for our patients. We began by highlighting the physical health benefits of exercise and dance to encourage participation. Throughout the intervention, we employed a gradual progression, starting with simple movements and increasing complexity to ensure accessibility and maintain interest. Instructors provided clear guidance and offered continuous encouragement to promote active involvement during each session. Additionally, we introduced small rewards, such as snacks, after each session to further motivate patients and enrich their experience. We believe these strategies will foster greater engagement and create a more enjoyable experience in the dance intervention. We recorded the attendance of each group of subjects.

1. Dancer intervention process

As illustrated in Figure A.1(A), the intervention was conducted in two distinct stages. In the initial stage, dance movements characterized by strong rhythmicity were selected, with an emphasis on enhancing the patients' fundamental motor skills. In the subsequent stage, dance movements involving complex combinations and variability were chosen to enhance the patients' capacity to integrate intricate motor patterns. Both stages incorporated movement observation and imitation, rhythmic auditory perception, spatial navigation, and the perception of relative body positioning. Throughout the process, the dance intervention placed a strong emphasis on the observation, perception, and imitation of dance movements. The patients' mastery of dance movements was considered when evaluating the final training outcomes.

1. Aerobic exercise intervention process

To precisely investigate the characteristics of dance, we compared it with an aerobic exercise group to extract dance-specific features. The aerobic exercise group was matched the exercise volume of the dance intervention while avoiding complex skill acquisition and minimizing cognitive load (Figure A.2). The movements in the aerobic exercise regimen: (1) were derived from everyday activities and did not require additional cognitive effort; (2) have been employed as aerobic stimuli in prior studies; (3) were safe and appropriate for the patients, with no risk of potential injury.


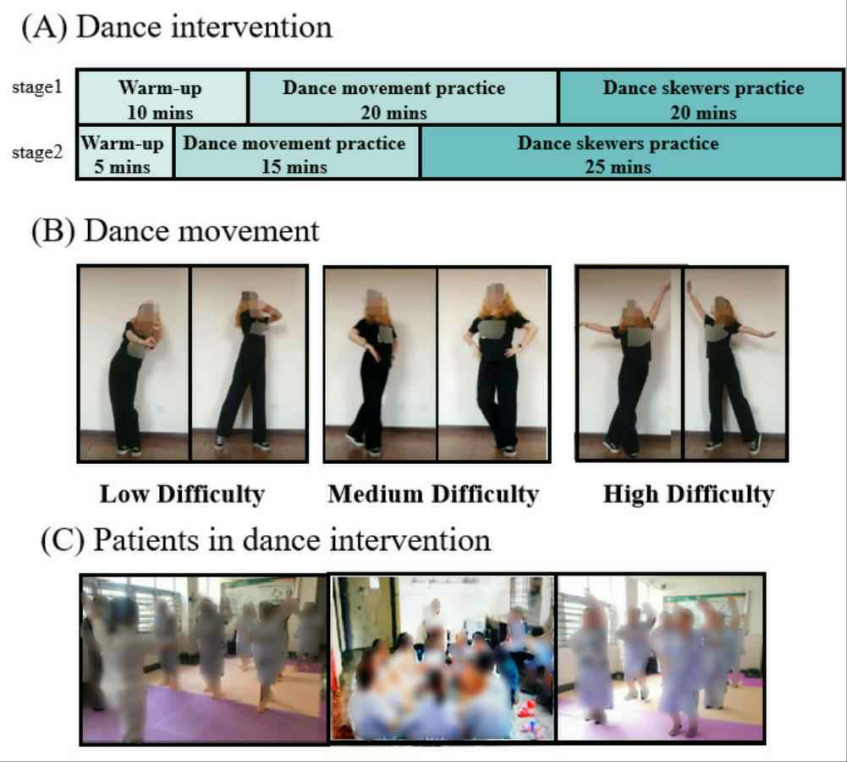


*Figure A. 1 Dance intervention program. (A)Two stages of dance intervention procedure. (B)Dance movement examples used in dance intervention. (C)Patients were practising dance movements under the guidance of the lead nurse.*


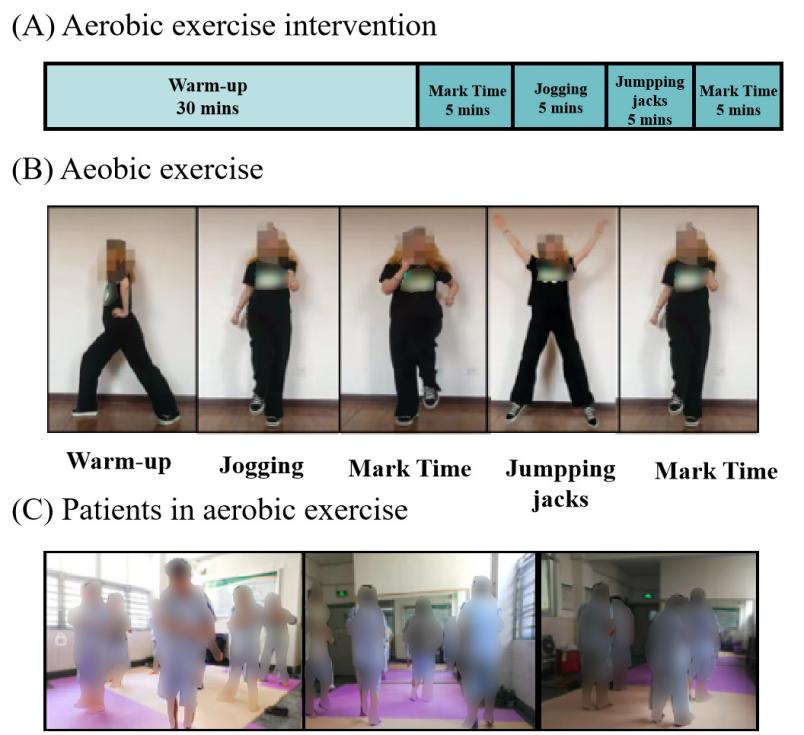


Figure A. 2. Aerobic exercise intervention program (A)Aerobic exercise intervention content. (B)Several movements in aerobic exercise intervention. (C)Patients was practicing aerobic exercise movements under the guidance of the lead nurse.

**Supplementary Material B**

Heart rate (HR) was recorded before, during, and after each intervention session. The target HR range for moderate-intensity exercise in patients with schizophrenia was set at 110–140 beats per minute, calculated using the Karvonen formula based on maximal heart rate (220−age). Exercise volume was estimated using VO_2_ max, and the Karvonen formula was applied to determine each patient’s target HR. However, due to patients’ physical conditions and performance levels, the actual HR during the interventions were approximately 80–120 bpm. Although a small proportion of patients did not reach the target range, no significant difference in HR was observed between the dance intervention group and the aerobic exercise group. The lower-than-expected HR may reflect the patients’ cardiovascular vulnerabilities. The formula is as follows.

$$\text{Target Heart Rate=[(}\max\text{HR-resting HR) ×}\text{(40\% to 50\%)}\text{Intensity] + resting HR }\text{（}\text{1}\text{）}$$

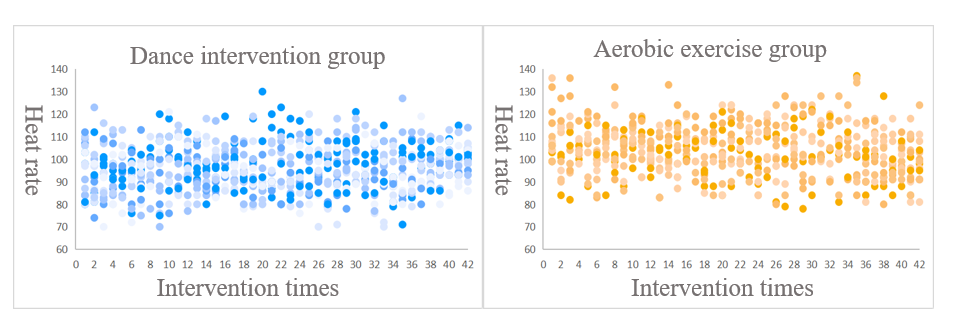


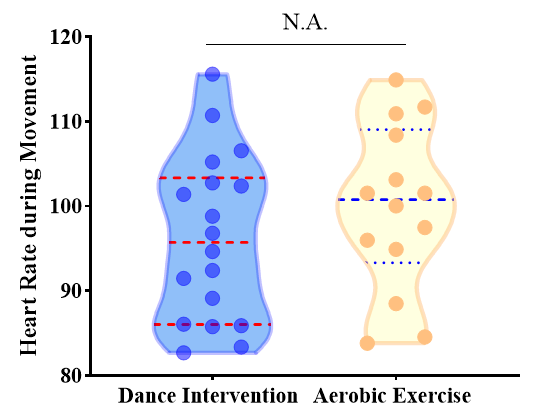


*Figure B. 3 The heart rate during interventions*

**Supplementary Material C**

Table C.1 Clinical symptom and Cognitive function of patients at baseline

|  | Dance intervention  group | Aerobic exercise  group | $p$ |
| --- | --- | --- | --- |
| PANSS | 61.26 (7.45) | 61.20 (9.07) | 0.67 |
| NOISE | 67.15 (11.95) | 61.33 (7.32) | 0.36 |
| TMT | 21.37 (7.06) | 23.21 (6.05) | 0.88 |
| SC | 21.53 (10.80) | 18.41 (12.53) | 0.55 |
| CF | 28.00 (8.58) | 32.46 (7.54) | 0.24 |
| SS | 25.00 (12.30) | 23.77 (13.83) | 0.60 |
| DS | 29.56 (14.75) | 26.69 (11.86) | 0.92 |
| BVMT-R | 30.56 (13.32) | 25.15 (9.51) | 0.28 |
| HVLT-R | 28.53(14.97) | 35.29 (7.97) | 0.65 |
| CPT-IP | 28.18 (8.44) | 34.92 (9.94) | 0.13 |
| NAB Mazes | 23.40 (11.26) | 14.67 (15.17) | 0.15 |
| MEST | 21.93 (11.59) | 20.54 (9.32) | 0.372 |

*Indicated values are shown as mean (standard deviation). PANSS: Positive and Negative Symptom Scale; NOISE: Nurses' Observation Scale for Inpatients Evaluation; TMT: Trail Making Test; SC: Symbol Coding; CF: Category Fluency; CPT-IP, Continuous Operation Test-identical pairs; SS: Spatial Span; DS, Digit Span Test; HVLT-R: Hopkins Word Learning Test-Revised; BVMT-R: Brief Visuospatial Memory Test-Revised; NAB Mazes: Neuropsychological Assessment Battery Mazes; MEST, Mayer-Salovey-Caruso Emotional Intelligence Quotient Test; p values for the comparisons (two-sample t-tests) between dance group and aerobic exercise group; *p<0.05, **p<0.01 and ***P<0.001.*

**Supplementary Material D**

Table D.2 Repeated ANOVA results on clinical assessment

|  |  | Dance intervention  group | Aerobic exercise  group | Time  effect  p value | Group  effect  p value | Interaction  effect  p value |
| --- | --- | --- | --- | --- | --- | --- |
| PANSS-P | baseline | 9.28(2.99) | 8.57(1.91) | 0.34 | 0.38 | 0.34 |
|  | 3 months | 9.28(3.27) | 8.29(1.98) |  |  |  |
| PANSS-N | baseline | 21.74 (5.14) | 23.93 (5.81) | 0.001^**^ | 0.58 | 0.58 |
|  | 3 months | 19.74 (5.17) | 21.27 (6.15) |  |  |  |
| PANSS-G | baseline | 29.35(3.10) | 28.07(5.14) | 0.63 | 0.89 | 0.17 |
|  | 3 months | 27.41(3.43) | 29.00(5.01) |  |  |  |
| PANSS-total | baseline | 61.26 (7.45) | 61.20 (9.07) | 0.003^**^ | 0.93 | 0.73 |
|  | 3 months | 58.05 (9.50) | 58.60 (9.44) |  |  |  |
| NOISE | baseline | 67.15 (11.95) | 61.33 (7.32) | <0.001^***^ | 0.14 | 0.14 |
|  | 3 months | 55.94 (6.41) | 53.66 (5.92) |  |  |  |

Indicated values are shown as mean (standard deviation). PANSS: Positive and Negative Symptom Scale; PANSS-P: the positive scores of PANSS; PANSS-N: the negative scores of PANSS; PANSS-G: the general scores of PANSS; NOISE: Nurses' Observation Scale for Inpatients Evaluation; *p<0.05, **p<0.01 and ***P<0.001.

Table D.3 Repeated ANOVA results on cognitive function measured by MCCB

| **Cognitive domain** | **Test** |  | **Dance interventiongroup** | **Aerobic exercisegroup** | **Time effectp value** | **Group effectp value** | **Time×Group interaction effect p value** |
| --- | --- | --- | --- | --- | --- | --- | --- |
| Processing speed | TMT | baseline | 21.37 (7.06) | 23.21 (6.05) | 0.003^**^ | 0.59 | 0.62 |
|  |  | 3 months | 24.68 (8.69) | 25.57 (9.52) |  |  |  |
|  | SC | baseline | 21.53 (10.80) | 18.41 (12.53) | 0.06 | 0.25 | 0.1 |
|  |  | 3 months | 26.26 (12.94) | 18.75 (12.74) |  |  |  |
|  | CF | baseline | 28.00 (8.58) | 32.46 (7.54) | 0.001^**^ | 0.144 | 0.8 |
|  |  | 3 months | 33.31 (7.94) | 37.38 (9.84) |  |  |  |
| Working memory | SS | baseline | 25.00 (12.30) | 23.77 (13.83) | 0.001^**^ | 0.88 | 0.76 |
|  |  | 3 months | 31.94 (12.60) | 31.92 (11.87) |  |  |  |
|  | DS | baseline | 29.56 (14.75) | 26.69 (11.86) | 0.006^**^ | 0.49 | 0.71 |
|  |  | 3 months | 33.31 (11.24) | 32.77 (13.94) |  |  |  |
| Visual learning | BVMT-R | baseline | 30.56 (13.32) | 25.15 (9.51) | 0.006^**^ | 0.021^*^ | 0.8 |
|  |  | 3 months | 34.31 (13.17) | 28.00 (15.65) |  |  |  |
| Verbal learning | HVLT-R | baseline | 19.20 (15.51) | 9.34 (6.05) | 0.015^*^ | 0.13 | 0.019^*^ |
|  |  | 3 months | 34.00 (10.91) | 20.73 (13.47) |  |  |  |
| Attention/Vigilance | CPT-IP | baseline | 19.41 (9.77) | 22.50 (12.31) | 0.22 | 0.98 | 0.026^*^ |
|  |  | 3 months | 24.23 (10.03) | 21.00 (9.40) |  |  |  |
| Reasoning and Problem-solving | MAZE | baseline | 23.40 (11.26) | 14.67 (15.17) | 0.91 | 0.266 | 0.95 |
|  |  | 3 months | 24.60 (16.48) | 15.00 (11.0) |  |  |  |
| Social cognition | MEST | baseline | 21.93 (11.59) | 20.54 (9.32) | 0.58 | 0.37 | 0.43 |
|  |  | 3 months | 22.40 (8.49) | 17.81 (6.46) |  |  |  |

*Indicated values are shown as mean (standard deviation). TMT: Trail Making Test; SC: Symbol Coding; CF: Category Fluency;* *CPT-IP:* *Continuous Operation Test-identical pairs; SS: Spatial Span; DS, Digit Span Test; HVLT-R: Hopkins Word Learning Test-Revised; BVMT-R: Brief Visuospatial Memory Test-Revised; MAZES: Psychological Assessment Test Package (NAB) Maze; MEST, Mayer-Salovey-Caruso Emotional Intelligence Quotient Test; ∗p<0.05, ∗∗p<0.01 and ***P<0.001*
